# Negative autoregulation of BMP dependent transcription by SIN3B splicing reveals a role for RBM39

**DOI:** 10.1038/srep28210

**Published:** 2016-06-21

**Authors:** Noel Faherty, Matthew Benson, Eshita Sharma, Angela Lee, Alison Howarth, Helen Lockstone, Daniel Ebner, Shoumo Bhattacharya

**Affiliations:** 1 Division of Cardiovascular Medicine, Radcliffe Department of Medicine, University of Oxford, Oxford, United Kingdom; 2 Wellcome Trust Centre for Human Genetics, University of Oxford, Oxford, United Kingdom; 3 Nuffield Department of Medicine, University of Oxford, Oxford, United Kingdom; 4 Target Discovery Institute, University of Oxford, Oxford, United Kingdom

## Abstract

BMP signalling is negatively autoregulated by several genes including SMAD6, Noggin and Gremlin, and autoregulators are possible targets for enhancing BMP signalling in disorders such as fibrosis and pulmonary hypertension. To identify novel negative regulators of BMP signalling, we used siRNA screening in mouse C2C12 cells with a BMP-responsive luciferase reporter. Knockdown of several splicing factors increased BMP4-dependent transcription and target gene expression. Knockdown of RBM39 produced the greatest enhancement in BMP activity. Transcriptome-wide RNA sequencing identified a change in *Sin3b* exon usage after RBM39 knockdown. SIN3B targets histone deacetylases to chromatin to repress transcription. In mouse, *Sin3b* produces long and short isoforms, with the short isoform lacking the ability to recruit HDACs. BMP4 induced a shift in SIN3B expression to the long isoform, and this change in isoform ratio was prevented by RBM39 knockdown. Knockdown of long isoform SIN3B enhanced BMP4-dependent transcription, whereas knockdown of the short isoform did not. We propose that BMP4-dependent transcription is negatively autoregulated in part by SIN3B alternative splicing, and that RBM39 plays a role in this process.

Members of the BMP/TGFβ superfamily play critical roles in pluripotency, embryonic stem cell differentiation and developmental patterning^1^. BMP signalling is required for heart development^2^ and cardiovascular homeostasis^3^. Signalling by BMP ligands occurs via binding to type I/II serine/threonine kinase receptors^4^. BMPs signal via the type II receptor (BMPR2) and via activin type II receptors. Type I BMP receptors form two groups, BMPR1A/R1B (ALK3/6) and the ALK1/2 group^5^. A number of co-receptors have been identified – among these endoglin (ENG) and betaglycan play roles in vascular disease^6^. BMP receptors phosphorylate receptor SMADs (SMAD1/5), which associate with the co-SMAD4 and translocate to the nucleus. These complexes bind specific elements, including the *ID* genes, which are negative regulators of cell differentiation and positive regulators of cell proliferation^7^. BMP signalling is negatively autoregulated by SMAD6, which is induced by BMP ligands^8^ and competes with SMAD1/5 for co-SMAD4^9^. BMP ligands also induce antagonists such as Gremlin, BAMBI and BMPER in an autoregulatory manner^10^,^11^. In cardiac development, a SMAD1/NKX2-5 negative feedback loop induced by BMP2 is required for progenitor specification^12^.

Reduction in BMP signalling is a causal mechanism in certain disorders. Haploinsufficient mutations in *BMPR2* are found in 80% of familial and between 15–40% of idiopathic cases of pulmonary arterial hypertension^5^,^13^. Deficiency of BMPR2 expression is also reported in non-genetic forms of pulmonary arterial hypertension^14^. The balance between BMP and TGFβ signalling is also thought to be important in fibrosis^15^. BMP7 has anti-fibrotic activity in cardiac^16^ and renal^17^ models, demonstrating proof-of-concept that activating BMP signalling may be useful. Targeting negative feedback is a potential strategy for enhancing BMP signalling. Here, we used siRNA screening to identify novel negative regulators of BMP signalling. We identified a subset of RNA binding/mRNA splicing factors, including RBM39, which negatively regulate BMP4-dependent transcription. We show that alternative splicing of the transcriptional repressor SIN3B is induced by BMP4 and can inhibit transcription. We find RBM39 is necessary for this alternative splicing of SIN3B. We propose that BMP4-dependent transcription is negatively autoregulated in part by SIN3B splicing, and that RBM39 plays a role in this process.

## Results

### High-throughput siRNA screen identifies splicing factors negatively regulating BMP4-dependent transcription

**Figure 1:**
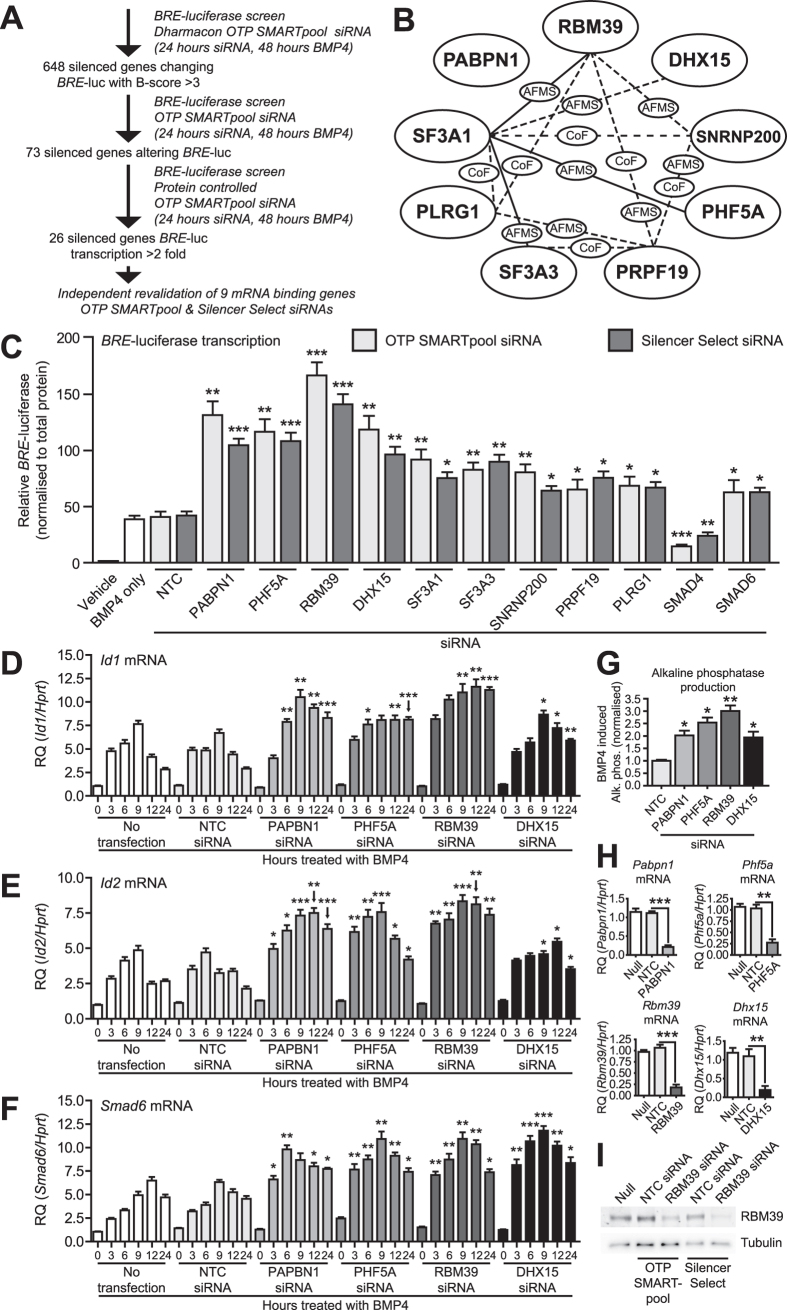
Identification of genes regulating BMP4-dependent transcription and gene expression using high-throughput siRNA screening. (**A**) Schematic diagram of high-throughput siRNA screen and target validation of ~10000 genes in mouse C2C12 myoblast cells using the BMP responsive *BRE*-luciferase reporter. (**B**) Interactome network of identified mRNA binding/splicing factors, from BioGRID^56^, based on several published studies. Solid lines indicate interactions reported in mouse, dashed lines indicate interactions reported in human. No interactions are reported between PABPN1 and the other splicing factors. CoF, co-fractionation, AFMS, affinity capture-mass spectrometry. (**C**) Luciferase assay in mouse C2C12 cells containing an integrated *BRE*-luciferase reporter. Cells were transfected with ON-TARGETplus (OTP) SMARTpool or Silencer Select siRNAs for 24 hours followed by treatment with BMP4 for 24 hours, with the exception of a control treated with diluent only (Vehicle). Knockdown of SMAD4 and SMAD6 (to inhibit and enhance BMP4-dependent transcription, respectively) were used as negative and positive controls for assay activity, respectively. Y-axis shows relative luciferase activity (mean +/− S.E.M., n = 3) normalised for total cellular protein. *P < 0.05, **P < 0.01, ***P < 0.001, all versus non-target control siRNA (NTC). (**D**–**F**) *Id1*, *Id2* and *Smad6* qPCR. C2C12 cells were transfected with OTP SMARTpool siRNAs for 24 hours followed by treatment with BMP4 for the hours indicated. Y-axis shows relative target gene expression (RQ, mean +/− S.E.M., n = 3) normalised to the housekeeping gene *Hprt*. *P < 0.05, **P < 0.01, ***P *<* *0.001* versus each graphs respective non-target control (NTC) siRNA at the same timepoint. (**G**) Alkaline phosphatase expression in C2C12 cells. Cells were transfected with siRNA for 24 hours, followed by treatment with BMP4 for 72 hours. Y-axis shows BMP4 induced alkaline phosphatase (mean +/− S.E.M., n = 3), normalised to total protein. *P < 0.05, **P < 0.01, all versus NTC siRNA. (**H**) *Pabpn1*, *Phf5a*, *Rbm39* and *Dhx15* qPCR in C2C12 cells. Cells were transfected with siRNAs for 24 hours. Y-axis shows relative target gene expression (RQ, mean +/− S.E.M., n = 3) normalised to the housekeeping gene *Hprt*. **P < 0.01, ***P < 0.001. (**I**) RBM39 protein levels in C2C12 cells. Cells were transfected with siRNA for 48 hours. Representative blot for n = 2 experiments shown.

We used C2C12 mouse myoblast cells containing a BMP-response element luciferase (*BRE-*luc) transcriptional reporter. We screened 9968 genes (Fig. 1A) using pooled siRNA to identify those, that when knocked down, enhanced BMP4-dependent transcription and identified 648 siRNAs that changed *BRE*-luc activity with a B-score > 3. To control for variation in cell number, we re-screened these hits, controlling for total cellular protein, and identified 26 siRNAs activating *BRE-*luc > 2-fold. A major group encoded RNA binding/mRNA splicing proteins, most of which are reported to interact (Fig. 1B). These targets have RNA-binding properties and play roles in the spliceosomal machinery (Supplementary Table S1). To confirm reproducibility, we repeated knockdown with original pooled siRNA and independently derived siRNAs from an alternate source. With both siRNAs, knockdown of these splicing factors increased *BRE-*luc in response to BMP4 (Fig. 1C).

### Knockdown of PABPN1, PHF5A, RBM39 and DHX15 enhances BMP4-dependent target gene expression

Knockdown of four splicing factors (PABPN1, PHF5A, RBM39 and DHX15) produced the strongest increase in *BRE*-luc transcription. We next explored the role of these factors in regulating expression of the endogenous BMP signalling target genes ID1, ID2, and SMAD6. We examined induction of *Id1*, *Id2* and *Smad6* mRNA in response to BMP4 over a 24 hour period and found that knockdown of PABPN1, PHF5A, RBM39 or DHX15 resulted in increased expression of these target genes, which was detectable by 3/6 hours and sustained at 24 hours (Fig. 1D–F). While knockdown of any splicing factor was sufficient to sustain increased *Id1*/*Id2* expression at 24 hours, the dynamics of increased expression varied depending on the splicing factor inhibited. Knockdown of RBM39 produced the earliest and most durable increase, while knockdown of PABPN1 and DHX15 showed a time dependent return towards basal levels. Knockdown of PHF5A produced a time dependent increase in *Id2* expression, but its effect on *Id1* was more distinct, where the increase plateaued after 6 hours of BMP4. This level was then sustained to 24 hours. This observation suggests that there may be a rate-limiting intermediate involved in this effect, limiting enhanced expression of *Id1* after PHF5A knockdown. Knockdown of all four splicing factors produced similar dynamics of increased expression of *Smad6* mRNA in response to BMP4. We also measured production of alkaline phosphatase, a BMP target gene, in C2C12 cells^18^. Depletion of PABPN1, PHF5A, RBM39 and DHX15 increased alkaline phosphatase production over 3 days in response to BMP4 (Fig. 1G). In these experiments we achieved mRNA knockdown of ~75–80% (Fig. 1H). These results were re-confirmed using alternate siRNA (Supplementary Fig. S1). Knockdown of RBM39 produced the strongest enhancement of BMP4-dependent transcription, target gene expression and alkaline phosphatase. Knockdown of RBM39 with siRNA also decreased RBM39 protein (Fig. 1I). To evaluate if the effect of RBM39 knockdown occurred at the initial level of activation of BMP signalling, we examined phosphorylation and localisation of SMAD1/5. Knockdown of RBM39 did not affect SMAD1/5 phosphorylation or nuclear localisation in response to BMP4 (Supplementary Figs. S2 and S3). These results suggested that RBM39 acts downstream of the SMAD signalling response, at transcriptional or post-transcriptional levels.

### Transcriptome-wide exon analysis after RBM39 knockdown suggests regulation of SIN3B

**Figure 2:**
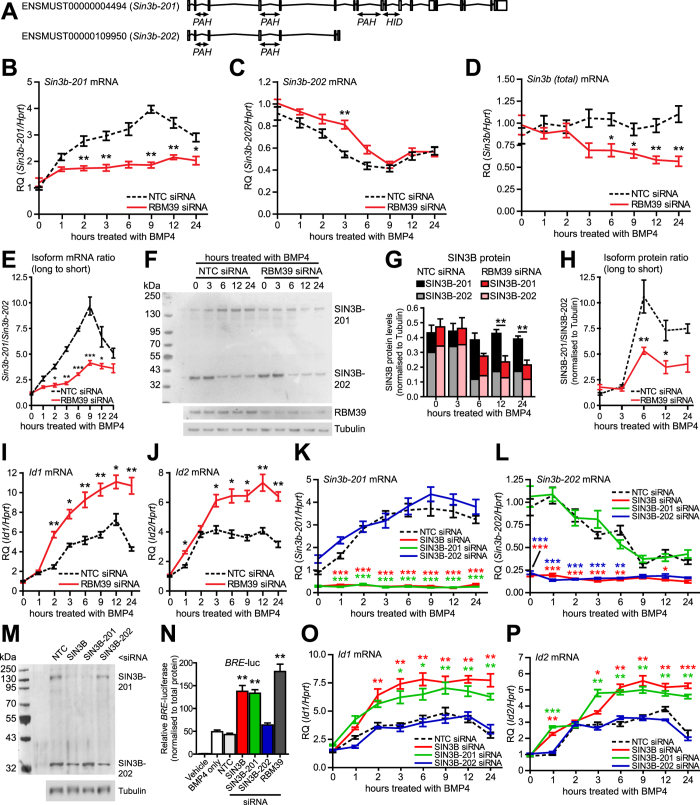
Expression of alternative *Sin3b* splice isoforms in mouse C2C12 cells; effect of knockdown of specific isoforms on BMP4-dependent transcription. (**A**) Mouse *Sin3b* is alternatively spliced as one of two isoforms. Translated *Sin3b-201* contains both paired amphipathic helix (PAH) and histone deacetylase interacting (HID) domains. *Sin3b-202* contains only PAH domains. C2C12 cells were transfected with either RBM39 siRNA or non-target control (NTC) siRNA for 24 hours followed by treatment with BMP4. (**B–D**) *Sin3b-201*, *Sin3b-202* and total *Sin3b* qPCR. Y-axis shows relative expression (RQ, mean +/− S.E.M., n = 3) normalised to *Hprt*. *P < 0.05, **P < 0.01, versus NTC at same timepoint. (**E**) Ratio of *Sin3b* mRNA levels. Y-axis shows averaged ratio for n = 3 experiments (mean +/− S.E.M) *P < 0.05, **P < 0.01, ***P < 0.001, versus NTC at the same timepoint. (**F**) Expression of SIN3B in C2C12 cells. Representative blot for n = 3 experiments shown. (**G**) Quantification of SIN3B isoforms. Y-axis shows expression normalised to tubulin. **P < 0.01. (**H**) Ratio of SIN3B-201 to SIN3B-202. Y-axis shows averaged ratio for n = 3 experiments (mean +/− S.E.M.). *P < 0.05, **P < 0.01, versus NTC at the same timepoint. (**I,J**) *Id1* and *Id2* qPCR. Y-axis shows relative expression (RQ, mean +/− S.E.M., n = 3) normalised to *Hprt*. *P < 0.01, **P < 0.01, versus NTC siRNA at same timepoint. (**K,L**) *Sin3b-201* and *Sin3b-202* qPCR. Cells were transfected with siRNA to total SIN3B, SIN3B-201 or SIN3B-202 for 24 hours, followed by treatment with BMP4. Y-axis shows relative expression (RQ, mean +/− S.E.M., n = 3) normalised to *Hprt*. *P < 0.05, ***P < 0.001, versus NTC at the same timepoint. (**M**) Expression of SIN3B in C2C12 cells. Cells were transfected with siRNA for 48 hours. Representative blot of n = 2 experiments. (**N**) C2C12 *BRE*-luc cells were transfected with siRNA for 24 hours, followed by treatment with BMP4 for 24 hours. Y-axis shows relative *BRE*-luciferase (mean +/− S.E.M., n = 3). **P < 0.01 versus NTC. (**O,P**). *Id1* and *Id2* qPCR. Cells were transfected with siRNA for 24 hours, followed by treatment with BMP4. Y-axis shows relative expression (RQ, mean +/− S.E.M., n = 3) normalised to *Hprt*. *P < 0.05, **P < 0.01, ***P < 0.001, versus NTC at the same timepoint.

RBM39 (also known as CAPER-alpha and HCC1), encodes an RNA-binding protein of the U2AF65 small nuclear ribonucleoprotein family. RBM39 splices VEGF isoforms in metastatic breast cancer cells and loss of RBM39 in sarcoma cells alters VEGF isoform ratio towards more mitogenic/angiogenic forms of VEGF^19^. To explore if RBM39 affects splicing in mouse cells, we knocked down mouse RBM39 and found that *Vegfa* mRNA isoform expression was altered in C2C12 cells (Supplementary Fig. S4), indicating cross species conservation. To identify splicing targets of RBM39 that could have a functional role in the BMP/TGFβ pathway, we used transcriptome-wide RNA-sequencing after siRNA knockdown of RBM39 (Supplementary Fig. S5). Analysis of differential exon usage found multiple targets with altered exon expression in the BMP/TGFβ pathway (Supplementary Fig. S6). Of these potential targets, we observed a change in splice variant expression of the transcriptional regulator *Sin3b* (Supplementary Fig. S7), a reported co-repressor of TGFβ superfamily signalling^20^. Gene expression analysis from sequencing data indicated that expression of *Sin3b* was not substantially changed (Supplementary Fig. S7). In mouse, *Sin3b* is alternatively spliced to generate ‘long’ and ‘short’ isoforms, with the short isoform lacking a histone deacetylase-interacting domain (Fig. 2A)^21^. Knockdown of RBM39 produced a shift in expression towards *Sin3b-201*, the longer isoform. This result was surprising as it would be predicted that if long isoform SIN3B was recruited to BMP target genes, it would repress, not enhance, BMP4-dependent transcription.

### BMP4 regulates expression of SIN3B alternative splice isoforms in mouse cells, which depends on RBM39

Sequencing was carried out without BMP4 signalling activation to examine the general splicing activity of RBM39. To clarify the link between BMP4, RBM39 and the *Sin3b* gene, we explored the effect of BMP4 on *Sin3b* splicing by isoform specific qPCR. *Sin3b-201* was increased in a time-dependent response to BMP4, with the response being detected by 1 hour, and peaking at 9 hours (Fig. 2B). In contrast, expression of *Sin3b-202* decreased, with the response being detected by 3 hours and peaking at 9 hours (Fig. 2C). The response of *Sin3b-201* to BMP4 was almost completely abrogated by RBM39 knockdown (Fig. 2B). However the response of *Sin3b-202* was not affected by RBM39 knockdown (Fig 2C). We also measured total levels of *Sin3b* mRNA, which did not alter significantly over time in response to BMP4 (Fig. 2D). When RBM39 was knocked down, total *Sin3b* mRNA declined over time after treatment with BMP4. We compared the ratio of *Sin3b-201* mRNA to *Sin3b-202* mRNA and found that knockdown of RBM39 prevented a time dependent change in ratio towards *Sin3b-201* induced by BMP4 (Fig. 2E). Knockdown of RBM39 was confirmed by qPCR (Supplementary Fig. S8). At the protein level, we observed that BMP4 increased SIN3B-201 expression by 6 hours, which persisted at 24 hours (Fig. 2F). SIN3B-202 protein expression reduced by 6 hours, persisting at 24 hours. The delay in SIN3B-201 protein change is consistent with the time taken for protein synthesis. As observed for mRNA, the increase in SIN3B-201 protein in response to BMP4 was almost completely abrogated by RBM39 knockdown. We quantified protein changes by densitometry (Fig. 2G). As with mRNA, knockdown of RBM39 prevented a BMP4-dependent change in ratio towards long SIN3B-201 (Fig. 2H). Total SIN3B protein expression remained relatively constant when cells were treated with BMP4, but knockdown of RBM39 resulted in a decrease in overall SIN3B over time. This decrease in total SIN3B can be accounted for by loss of SIN3B-202 mRNA and protein without a parallel increase in SIN3B-201. We determined the effect of BMP4 and RBM39 knockdown on expression of *Id1* and *Id2* genes at the same timepoints (Fig. 2I,J). BMP4 increased *Id1* expression by 2 hours; this peaked at 12 hours with a subsequent decline. An increase in *Id2* peaked after 3 hours and persisted to 12 hours with subsequent decline. These dynamics are broadly consistent with previous reports in C2C12 cells and elsewhere^22^. Knockdown of RBM39 significantly affected the response of *Id1* and *Id2* to BMP4. Specifically, there was an overall increased level of response, a delay in the *Id2* peak and increased expression of *Id1* persisting at 24 hours.

### Modulation of specific splice variants of SIN3B regulates BMP4-dependent transcription and target gene expression in mouse

Due to the histone deacetylase domain difference between SIN3B-201 and SIN3B-202, we hypothesised they may have different functional consequences for BMP signalling. We knocked down total SIN3B and specific transcripts of SIN3B with isoform specific siRNAs. We validated the isoform-specific siRNAs for specificity (Fig. 2K–M). Knockdown of total SIN3B or SIN3B-201, but not SIN3B-202, increased BMP4-dependent *BRE-*luc transcription (Fig. 2N). The enhancement of *BRE*-luc by total SIN3B knockdown or SIN3B-201 knockdown was almost as efficient as knocking down RBM39. We then examined if knockdown of these isoforms altered expression of BMP-target genes. Total SIN3B or SIN3B-201 knockdown increased BMP4-dependent *Id1* and *Id2* mRNA expression (Fig. 2O–P). The enhancement of *Id1* and *Id2* expression by total SIN3B or SIN3B-201 knockdown was broadly comparable with that caused knocking down RBM39. This suggests a majority of RBM39 action in negatively regulating BMP transcription and gene expression may occur via positive regulation of SIN3B-201.

### Knockdown of RBM39 increases BMP4-dependent transcription and target gene expression in human cells; expression of SIN3B alternative splice isoforms is also regulated by BMP4 and RBM39

**Figure 3:**
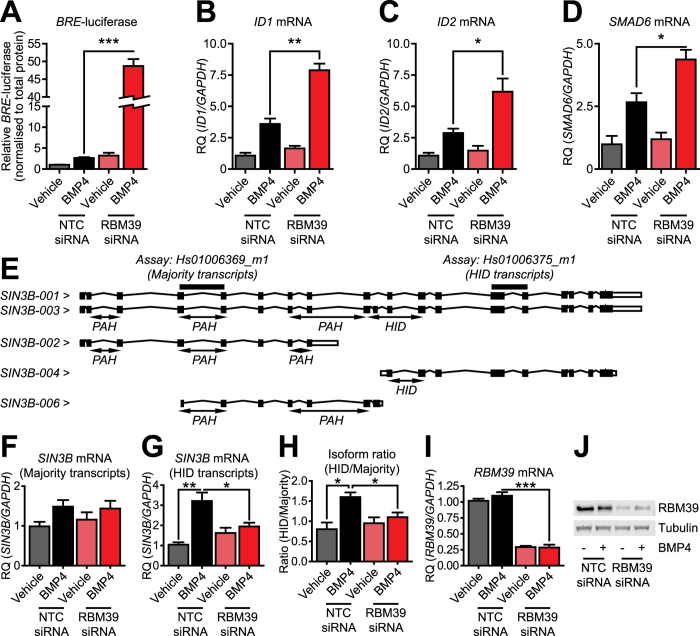
Effect of knockdown of RBM39 on BMP4-dependent transcription and SIN3B splicing in human U2OS cells. (**A**) Luciferase assay in U2OS osteosarcoma cells. Cells were co-transfected with OTP SMARTpool RBM39 siRNA or non-targeting control (NTC) siRNA and a *BRE*-luciferase reporter construct for 24 hours, followed by BMP4 treatment for 24 hours. Y-axis shows relative *BRE*-luciferase expression normalised to total protein (mean +/− S.E.M., n = 3). ***P < 0.001. (**B–D**) *ID1*, *ID2* and *SMAD6* qPCR. U2OS cells were transfected with RBM39 siRNA or NTC siRNA for 24 hours, followed by BMP4 treatment for 24 hours. Y-axis shows relative gene expression (RQ, mean +/− S.E.M., n = 3) normalised to the housekeeping gene *GAPDH*. *P < 0.05, **P < 0.01. (**E**) Schematic of specific transcripts measured by two different *SIN3B* qPCR gene expression assays. Human *SIN3B* is alternatively spliced to produce transcripts containing paired amphipathic helix (PAH) and histone deacetylase-interacting domains (HID). (**F**) Expression of the majority of human *SIN3B* transcripts by qPCR (gene expression assay Hs00396369_m1). Y-axis shows relative expression of transcripts (RQ, mean +/− S.E.M., n = 3) normalised to the housekeeping gene *GAPDH*. (**G**) Expression of human *SIN3B* transcripts containing the HID encoding exons by qPCR (gene expression assay Hs01006375_m1). Y-axis shows relative expression of transcripts (RQ, mean +/− S.E.M., n = 3) normalised to the housekeeping gene *GAPDH*. *P < 0.05, **P < 0.01. (**H**) Ratio of HID containing *SIN3B* transcripts to majority of *SIN3B* transcripts. Y-axis shows averaged ratio for n = 3 experiments (mean +/− S.E.M.). *P < 0.05. (**I**) Human RBM39 qPCR. Y-axis shows relative expression (RQ, mean +/− S.E.M., n = 3) normalised to the housekeeping gene *GAPDH*. ***P < 0.001. (**J**) Expression of RBM39 in U2OS cells. Representative blot of n = 2 experiments shown.

RBM39 is a splicing regulator (of VEGF) in human cells, and we observed that it spliced VEGF in mouse cells. To investigate if RBM39 also negatively regulates BMP4-dependent transcription in human cells, we transfected U2OS osteosarcoma cells with siRNAs to human RBM39 and a *BRE-*luc reporter. Knockdown of RBM39 increased *BRE-*luc in response to BMP4 (Fig. 3A). Knockdown of RBM39 in U2OS cells also increased expression of *ID1*, *ID2* and *SMAD6* mRNA levels in response to BMP4 (Fig. 3B–D). This demonstrates that negative regulation of BMP4-dependent transcription by RBM39 is not limited by species. We then examined if knockdown of RBM39 in human cells affected *SIN3B* splicing. Human *SIN3B* contains 5 mRNA transcripts (Fig. 3E). Similar to SIN3B in mouse, only some have histone deacetylase interacting domains. We used two gene expression assays to measure *SIN3B*, one which recognises the majority of transcripts, and one which only recognises those with the histone deacetylase-interacting domain. Expression of most *SIN3B* transcripts trended towards being responsive to BMP4, but did not reach significance (Fig. 3F). Expression of transcripts with the histone deacetylase-interacting domain was increased by BMP4 (Fig. 3G). We measured the ratio of induction of *SIN3B* transcripts containing a histone deacetylase interacting domain to the majority of *SIN3B* transcripts and found that BMP4 was sufficient to increase this ratio (Fig. 3H). Knockdown of RBM39 (Fig. 3I–J) attenuated both induction of histone deacetylase domain containing transcripts and the change in isoform ratio, suggesting that regulation of splicing of *SIN3B* by BMP4 via RBM39 can occur across species.

### RBM39 expression, localisation and phosphorylation is not regulated by BMP4

The experiments above indicated that SIN3B functions in an autoregulatory feedback loop to regulate BMP4-dependent transcription. We further investigated if BMP4 directly affects RBM39 function. BMP4 did not alter *Rbm39* mRNA expression, RBM39 protein or its nuclear localisation (Supplementary Fig. S9). RBM39 is likely to be phosphorylated (as it is homologous to SR proteins)^23^. We found that RBM39 is indeed phosphorylated, but this was not affected by BMP4 (Supplementary Fig. S9).

## Discussion

Augmentation of BMP signalling is a possible therapeutic strategy in pulmonary arterial hypertension and tissue fibrosis. In this study, we identified novel negative regulators of BMP signalling. We identified nine targets involved in mRNA splicing and processing, representing a small subset of over 200 individual proteins in the spliceosome^24^. Alternative splicing involves inclusion of specific exons and selection of splice sites to produce different mRNAs. The targets we identified play diverse roles in splicing and processing. Of these, knockdown of RBM39 produced the greatest enhancement in BMP activity. Initially characterised as a co-activator of AP-1 and the oestrogen receptor^25^, and apart from its role in VEGF splicing, RBM39 is also implicated in energy and redox homeostasis^26^ and cancer cell proliferation^27^.

After knockdown of RBM39 in mouse cells identified alternative splicing activity (of VEGF), we used transcriptome-wide sequencing to identify targets of RBM39, which identified SIN3B. The mammalian SIN3/HDAC complex comprises multiple subunits, including HDAC1, HDAC2, SAP18/30, SUDS3, RBBP4/7 and either SIN3A or SIN3B^28^. Both SIN3A and SIN3B contain multiple paired amphipathic helix (PAH) domains, which allow binding of transcription factors, and a histone deacetylase interacting domain (HID)^29^. SIN3 acts as a molecular scaffold to direct HDACs (bound via the HID domain) to specific sites and repress gene transcription^30^. The mSIN3/HDAC complex is reported to negatively regulate both BMP and TGFβ signalling^20^,^31^. SIN3B in mouse is spliced into long and short isoforms^21^ with both transcribed from a common transcriptional start site. While the long isoform contains a HID, the shorter isoform, SIN3B-202, lacks the HID and does not bind histone deacetylases^32^, but may repress by interacting with other transcription factors^32^.

RNA-sequencing identified a change after knockdown of RBM39, which increased the ratio of *Sin3b-201* to *Sin3b-202*. As we observed that knockdown of RBM39 increased BMP4-dependent transcription, and that SIN3B-201 was likely to possess greater repression than SIN3B-202 due to the presence of the HID, we explored this further. To clarify, we used isoform specific qPCR to measure both *Sin3b-201* and *Sin3b-202* levels in C2C12 cells. This was done with and without BMP4 and in the presence or absence of RBM39 knockdown. While we found an apparent trend for increased expression of *Sin3b-201* mRNA after RBM39 knockdown by qPCR (consistent with the results observed from RNA sequencing), the difference did not reach statistical significance. Instead we reproducibly found that BMP4 induced an increase in long SIN3B-201 mRNA and protein. This change was prevented by knockdown of RBM39. The difference in detected significance between sequencing data and qPCR may be a technology specific effect. RNA-sequencing provides a raw count of exon numbers compared to the relative expression by qPCR. As we do not have sequencing data from cells treated with BMP4, it is not possible to say what effect BMP4 treatment has on exon expression by sequencing. We speculate that because levels of *Sin3b-201* were not statistically different after RBM39 knockdown when measured by qPCR, data from sequencing may be representative of a (relatively) small change in low basal transcript levels that are amplified in terms of significance. It is, however, important to note that by both methods, RBM39 knockdown trends towards increasing *Sin3b-201*, at least at the mRNA level. Such an observation may suggest dual-activity of RBM39, where it slightly increases *Sin3b-201* in the absence of BMP4, but prevents BMP4 induced splicing in favour of *Sin3b-201*. In the latter part of this discussion, we refer to the likely importance of post-translational and complex-dependent regulation of RBM39. A future focus would need to investigate any potential for dual-activity arising from such regulation. In the interests of simplicity, our considerations below are limited to the validated splicing change mediated by RBM39 in the presence of BMP4.

Both *Sin3b-201* and *Sin3b-202* share a transcriptional start site. Therefore, the most likely explanation for the BMP4 induced regulation of *Sin3b* by RBM39 is that it occurs via alternative splicing rather than transcription. Knockdown of long SIN3B-201 enhanced transcription, while knockdown of short SIN3B-202 did not. These results indicate that BMP4-dependent transcription is negatively autoregulated in part by SIN3B alternative splicing. RBM39 knockdown prevented not only the increase in long SIN3B-201 induced by BMP4, but also the overall change in long-to-short isoform ratio. Due to limitations of pulling down RBM39, we have been unable to provide a direct physical link between RBM39 and *Sin3b* transcripts, e.g. using RNA immunoprecipitation.

We observed that the BMP4 induced change in *Sin3b* mRNA isoform expression occurred concurrently with increased expression of *Id1* and *Id2*. For both, increased expression preceded the change in SIN3B protein. This indicates that mechanisms other than altered *Sin3b* splicing are required for enhanced early induction of *Id1*/*Id2* after RBM39 knockdown. At 24 hours, the return of *Id1*/*Id2* expression towards basal levels coincided with sustained induction of long SIN3B-201 expression. Preventing splicing in favour of SIN3B-201 by knocking down RBM39 may therefore result in sustained induction of *Id1*/*Id2* in response to BMP4. The dynamics of BMP4-dependent *Id1*/*Id2* expression after knockdown of SIN3B or long SIN3B-201 were similar to those observed after knockdown of RBM39. This data supports long SIN3B-201 being the major isoform involved in the suppression of BMP signalling, and thus that the balance between isoforms is functionally important. Taken together, our results suggest that *Sin3b* splicing is a novel negative autoregulatory feedback mechanism induced by BMP4.

Our observations on the role of RBM39 in mediating BMP4 induced alternative splicing are complicated by finding that knockdown of RBM39 did not restore *Sin3b-202* mRNA. This was surprising, as there are only two described transcript isoforms of *Sin3b* in mouse. It may therefore be expected that a ratio should be maintained based on the observed change in *Sin3b* mRNA levels in cells treated with BMP4. Based on the actual observed changes, RBM39 is required for active splicing in favour of *Sin3b-201*, but not for BMP4-induced loss of *Sin3b-202*. It is important to note that the approach used here may be complicated by other post-transcriptional processes, including mRNA degradation, or post-translational modifications, which are speculated to regulate SIN3B function^30^. As the human *SIN3B* gene is more elaborately spliced, unknown splice variants in mouse *Sin3b* may also prevent simple interpretation. Further, our data indicates that in the presence of RBM39, total SIN3B mRNA and protein levels remain relatively static in response to BMP4. Knocking down RBM39 disrupts maintenance of SIN3B isoform ratios and produces a BMP4-dependent overall loss of SIN3B from basal levels. The two SIN3 isoforms, SIN3A and SIN3B, have both unique and shared gene targets^33^. It is therefore important to acknowledge that changes in total SIN3B expression after RBM39 knockdown may have elaborate causal effects depending on changes in overall mSIN3 complex activity.

Changes in cellular splicing programs in response to activated cell signalling cascades have been described and can occur through mechanisms including splicing factor synthesis/degradation, different protein-protein interactions and altered nuclear translocation (extensively reviewed elsewhere^34^). To examine the functional link between BMP and activity of RBM39, we characterised RBM39 expression in response to BMP4. BMP4 did not regulate *Rbm39* mRNA levels or RBM39 protein. RBM39 has an arginine/serine (RS) rich domain, similar to the SR family of splicing proteins which are phosphorylated in the RS domain^23^. Phosphorylation of SR and SR-like proteins is reported as a mechanism of linking extracellular signalling to alternative splicing activity^35^. We found that RBM39, which is classified as SR-like^23^, is natively phosphorylated, but this is not dependent on the activity of BMP4. This suggests that phosphorylation dependent activation of RBM39 in response to BMP4 is not required. Other post-translational mechanisms of regulation of SR proteins include acetylation and methylation^36^. Large-scale proteomic analysis has identified lysine acetylation in SR proteins, which may be target substrates for various histone acetyltransferases^37^. Arginine methylation has been detected on multiple RNA binding proteins near phosphorylation sites; hyper-methylation of such sites can block nuclear import and turnover^38^. While we have not investigated whether acetylation/methylation of RBM39 occurs in response to BMP4, we observed no apparent change in localisation of RBM39 induced by BMP4. SR and SR-like proteins are phosphorylated by the SR protein kinases (SRPKs) and CDC2-like kinases (CLKs), which affect their localisation and turnover^39^. RBM39 is reported to interact with SRPK2^40^. There may therefore be more subtle and dynamic variation in RBM39 localisation, which is not accounted for by our results. Importantly, TGFβ is reported to regulate alternative splicing of VEGF through a mechanism requiring both P38 MAPK signalling and CLK1/4 dependent SR protein phosphorylation^41^. We found that knockdown of RBM39 alters VEGF splicing, which raises the possibility of the existence of a similar non-canonical signalling axis regulated by BMP4. A more precise and comprehensive evaluation of RBM39 as an SR-like protein, in terms of its localisation and post-translational modification in response to BMP4, may provide more insight.

In addition to post-translational modifications, functional activity and specificity of mRNA splicing factors can be conferred by variation in interacting partners. RBM39 is reported to interact directly with U2AF65/U2AF2^42^, an essential component of the U2 major splicing complex. U2AF65 binds to polypyrimidine-rich sequences found near the 3′-end of most introns to promote stable U2 association with pre-mRNA^43^. U2AF65 and RBM39 have a number of shared interacting partners, including the principal SRPKs, multiple SR proteins and several auxiliary PRPF splicing proteins^44^. The recruitment and release of these factors to the U2 splicing complex is highly dynamic^45^. A question arising from our studies is if the dynamics and constituent components of this complex during pre-mRNA binding could be modified in response to BMP signalling. Of the known RBM39-interacting SR proteins, SF3B4/SAP49 can bind the type I BMP receptor BMPR1A/ALK3^46^. Overexpression of SF3B4 also suppresses *Id* reporter activity and negatively regulates cell surface levels of BMPR1A^47^. SF3B4 is reported to interact with SF3A1, SF3A3 and PHF5A^48^, all of which increase *BRE*-luciferase transcription in response to BMP4 when knocked down. Taken with the interactome observed for the 9 mRNA binding/splicing factors, there may be significant opportunity for overlap between these factors and other splicing factors in contributing to regulation of BMP4-dependent transcription. Intricate and temporary interactions between RBM39 and other splicing factors may be induced by, and required for, this regulation. Profiling such a network will require significant effort, but would be necessary to appreciate the intermediate steps between BMP pathway activation and RBM39 mediated splicing of SIN3B.

BMP/TGFβ superfamily signalling is made more complicated by the diversity of both ligand and receptor (comprehensively recently reviewed elsewhere with respect to cardiovascular development and disease)^49^. In this study, we report results using BMP4 as a ligand, which, along with BMP2, binds to BMPR2 and ACVR2A/2B type II receptors. Both ligands bind to the BMPR1A/R1B (ALK3/6) type I receptors, but not ALK1/ALK2 receptors. In contrast, BMP7, as the archetypical member of its group, can bind BMPR2 and ACVR2A type II receptors and all of ALK2/3/6 type II receptors. Our observations, not published here, indicate that knockdown of RBM39 also enhances *BRE*-luciferase and *Id* gene transcription in response to BMP2 and BMP7. This is of particular relevance with regard to the established importance of loss of BMPR2 and other receptors in cardiovascular disease: mutations in BMP receptor II (BMPR2)^50^ and in the BMP co-receptor endoglin or the receptor activated SMAD9 result in reduced BMP signalling and primary pulmonary hypertension^13^,^51^. In the case of BMPR2, the majority of these mutations cause nonsense, frameshift or splice-site defects, resulting in premature transcript termination^52^. In heritable pulmonary arterial hypertension, the ratio between two alternatively spliced BMPR2 transcripts, with a single exon difference, results in different disease penetrance^50^. This change in BMPR2 receptor ratio required activity of the SR protein SRSF2. We did not find altered BMP receptor splicing after RBM39 by RNA sequencing, but this does not preclude the possibility of it occurring similarly to SIN3B, when BMP signalling is active. It is important to appreciate that splicing of BMP receptors by the mRNA splicing factors may confer additional subtlety on the regulatory mechanism we have described here. Modulation of mRNA splicing factors to produce a ‘generalised’ increase in BMP signalling in response to several ligands may be beneficial where specific receptor loss blunts signalling responses to a specific subset of BMP ligands.

In human U2OS cells, we observed changes in transcripts that contained exons encoding the HID domain of human SIN3B. This change in transcript ratio of HID transcripts to most other transcripts was both BMP4-responsive and inhibited by siRNA knockdown of human RBM39. This indicates that regulation of BMP4 induced *SIN3B* alternate splicing by RBM39 can occur across species. Knockdown of RBM39 in U2OS cells also increased transcription in response to BMP4. The functional link between RBM39 and the BMP signalling pathway therefore appears to be present in both mouse and humans.

**Figure 4:**
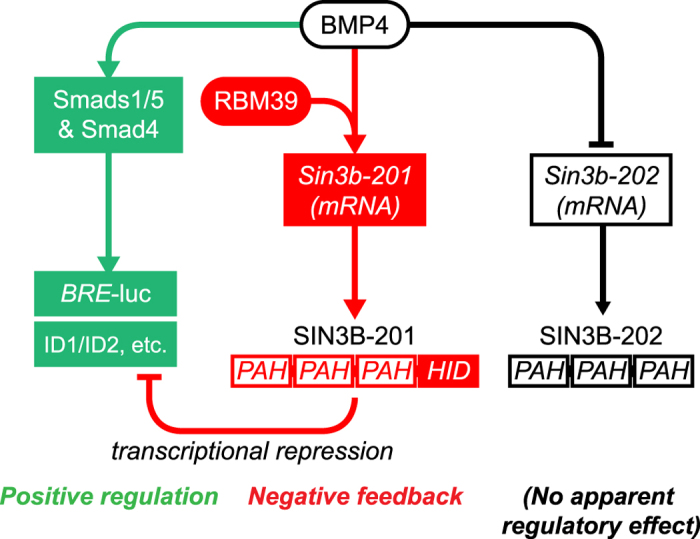
Proposed model of regulation of BMP signalling by alternative splicing of SIN3B via RBM39. BMP4 positively activates canonical signalling and transcription via Smad1/5 and Smad4 (green). In addition, BMP4 induces a switch in SIN3B isoform expression, which is dependent on the activity of RBM39. This can act as a negative feedback loop on BMP4-dependent transcription and gene expression through repression by SIN3B-201 (red). PAH, paired amphipathic helix domain, HID, histone deacetylase interacting domain.

In this study, we have found initial evidence that mRNA splicing factors provide a level of regulation of BMP signalling. As these factors are reported to interact with one another, their role in this regulation is likely complicated, and context specific. We have identified that RBM39 is required for BMP4 induced regulation of a transcriptional repressor of BMP signalling, and that different isoforms of mouse SIN3B have different regulatory effects on transcription induced by BMP4 (summarised in Fig. 4). This evidence indicates a novel negative autoregulatory mechanism, where SIN3B can be alternatively spliced in response to BMP4, and then negatively regulate BMP4-dependent transcription. Dissecting out the precise intermediate partners and mechanism of the BMP4/RBM39 response is a key outstanding question. This will depend on improved methods of interrogating splicing factors in complex with target mRNAs in different signalling contexts. Further research on the role of mRNA splicing factors in regulating BMP/TGFβ superfamily signalling may be therapeutically useful in disorders where enhancement of BMP signalling is a desired outcome.

## Methods

### Cell culture, vectors and transfections

Mouse myoblast C2C12 and human osteosarcoma U2OS (both from ATCC) were cultured in Dulbecco’s Modified Eagle’s Medium (DMEM) supplemented with 10% Foetal Bovine serum and 2 mM L-glutamine. The BMP reporter cell line *BRE*-luciferase C2C12 is previously described^53^. *BRE-*luciferase (hereafter *BRE-*luc) consists of two copies of the BMP responsive elements of the mouse *Id1* gene cloned into the pGL3 luciferase vector^54^. *BRE*-luc C2C12 cells were maintained in DMEM supplemented with 10% FBS, 2 mM L-glutamine and 700 μg/ml Geneticin/G418. U2OS cells were transiently transfected with pGL3 *BRE*-luc^54^ (50 ng per 96 well plate well) in Opti-MEM I (Gibco) using Fugene HD (Promega) to the manufacturer’s instructions. For *BRE*-luc assays (excluding the initial screen, detailed below), alkaline phosphatase expression and all qPCR, cells (stable C2C12 lines and transient U2OS) were transfected with siRNA using RNAiMax (Invitrogen) for 24 hours in DMEM with 10% FBS, L-Glutamine and then cultured with rhBMP4 (1 ng/ml, R&D Systems) in DMEM with 0.1% FBS and L-glutamine. For all experiments except timecourse data and expression of alkaline phosphatase, cells were treated with rhBMP4 for 24 hours. For alkaline phosphatase expression, cells were treated with rhBMP4 for 72 hours. For timecourse data, cells were cultured in DMEM with 0.1% FBS and L-glutamine for 24 hours before being treated with BMP4.

### Screen for enhancers of BMP4-dependent transcription

All liquid handling was performed using the Perkin Elmer Janus liquid handling platform unless noted. To create a customised siRNA library, we identified druggable genes in the Therapeutic Targets database^55^, INTERPRO protein families’ genes, and a list of druggable genes provided by GE Dharmacon. SiRNAs (ON-TARGETplus SMARTpool, to 9968 genes, GE Dharmacon) were diluted to 166.5 nM in a final volume of 60 μl in Opti-MEM. 20 μl of RNAiMax diluted 1:50 in Opti-MEM was added to the dilute siRNA and mixed. 18 μl of siRNA/RNAiMax mix was then transferred into duplicate 384-well plates. Non-targeting control and SMAD4 siRNA were plated, in addition to 2 wells of transfection reagent only. After a 25 min incubation at room temperature 2500 BRE-luc C2C12 cells in 72 μl of DMEM, 10% FBS, 2 mM L-Glutamine, were added to the transfection mix using a Flexdrop reagent dispenser (Perkin Elmer), to give a final concentration of 20 nM siRNA and 0.1 ul RNAiMAX, per well. The following day the transfection media was removed and replaced with media containing 0.1% FBS, L-glutamine and 2.5 ng/ml BMP4. After 48 hours incubation the media was removed and processed for luminescence using Steadylite (Perkin Elmer) as per manufacturer’s guidelines. Luminescence was measured using a BMG Polarstar plate reader. B-scores were calculated using Cell-HTS2 (http://web-cellhts2.dkfz.de/cellHTS-java/cellHTS2/). The average Z’ over all plates was 0.443.

### Validation and follow up of target genes

For validation and follow up of target genes, cells were transfected with 20 nM of siRNA for 96 well assays, scaled up to maintain this concentration in larger vessels. Initial siRNAs used were ON-TARGETplus SMARTpool from GE Dharmacon; siRNAs used were to PABPN1 (L-045150-01), PHF5A (L-014987-01), RBM39 (mouse L-048512-01, human L-011965-00), DHX15 (L-044844-01), SF3A1 (L-041779-01), SF3A3 (L-064034-01), PRPF19 (L-057330-01), PLRG1 (L-062980-01), SNRNP200 (L-041847-01) and SIN3B (total, L-042784-01). Custom siRNAs were picked to target only SIN3B-201 (sense 5′-UGGGAUAAGCAGAGAAAUUUU-3′) and SIN3B-202 (sense 5′-CUGCACAGCUGAGGAGUGAUU-3′). All changes were controlled by comparison to the effect of a non-target control (NTC) siRNA (D-001810-10). The effect of knockdown of several targets was further independently confirmed with Silencer Select siRNAs from Ambion; siRNAs used were to PABPN1 (s79501), PHF5A (s86709), RBM39 (s172699) and DHX15 (s64901). All changes were controlled against non-target control siRNA (4390843).

### Luciferase assays

After knockdown and treatment with BMP4, cells were lysed in luciferase lysis buffer (25 mM Tris-PO_4_, 2 mM CDTA, 1% Triton X-100). Lysates were assayed with luciferase assay buffer (25 mM glycyl glycine, 15 mM MgSO_4_, 15 mM KPO_4_, 4 mM EGTA, 2 mM ATP, 2 mM DTT, 100 μg/ml D-luciferin [Goldbio]). Total luciferase activity was normalised to cellular protein levels by BCA assay of lysate.

### RNA isolation, RNA sequencing, cDNA preparation and PCR/qPCR

Total RNA was isolated from cells using an RNeasy Micro kit (Qiagen) and converted to cDNA with a QuantiTect Reverse Transcription Kit (Qiagen) to the manufacturer’s instructions. Quantitative PCR (qPCR) was carried out using 2× Taqman Universal PCR Master Mix no-AmpErase UNG (Life Technologies) on a BioRad CFX96 qPCR system. Assays used (Life Technologies) were *Pabpn1* (Mm00479791_m1), *Phf5a* (Mm00466078_m1), *Rbm39/RBM39* (mouse Mm00504698_m1 and human Hs00863502_g1), *Dhx15* (Mm00492109_m1), *Id1/ID1* (mouse Mm00775963_g1 and human Hs03676575_s1), *Id2/ID2* (mouse Mm00711781_m1 and human Hs04187239_m1), *Smad6/SMAD6* (mouse Mm00484738_m1 and human Hs00178579_m1), *Sin3b-201* (Mm01249269_m1), *Sin3b-202* (Mm01247120_g1), total *Sin3b* (Mm01247119_m1) and human *SIN3B* (Hs01006369_m1 and Hs01006375_m1); all qPCR data was normalised to either *Hprt* (Mm01545399_m1) for mouse or *GAPDH* (Hs02758991_g1) for human samples and changes analysed using the ddCt method. PCR for *Vegfa* was carried out using IMMOLASE Taq polymerase (Bioline); primers used to detect alternative *Vegfa* isoforms were forward 5′-GCTGTAACGATGAAGCCCTG-3′, reverse 5′-CCGCCTTGGCTTGTCACA-3′, altered *Vegfa* isoform expression was normalised to *Gapdh* using primers forward 5′-GAGAGTGTTTCCTCGTCCCG-3′ and reverse 5′- ACTGTGCCGTTGAATTTGCC-3′. *Vegfa* isoform PCR products were gel extracted and Sanger sequenced to confirm their identity. For RNA sequencing, total RNA was extracted from C2C12 cells after knockdown with RBM39 siRNA (or non-target control siRNA) for 24 hours and DNase-I treated. Libraries were prepared with a TruSeq Stranded mRNA Library Prep Kit (Illumina) and 100 bp paired-end sequenced on an Illumina Hi-Seq platform.

### Alkaline phosphatase

Alkaline phosphatase expression was determined with a Phospha-Light kit (Life Technologies). Cells were transfected with siRNA for 24 hours, followed by treatment with BMP4 for 72 hours. Cells were lysed in dilution buffer containing 0.2% Triton X-100 and assayed using the manufacturer’s instructions, with the exception of not adding buffer containing non-placental alkaline phosphatase inhibitors.

### Protein extraction and western blotting

Total protein was extracted from cells in RIPA buffer (50 mM Tris-HCl, 150 mM NaCl, 5 mM EDTA, 0.5% Na-deoxycholate, 0.1% sodium dodecyl sulphate) supplemented with a protease and phosphatase inhibitor cocktail; protein lysate was combined with SDS loading buffer (4% SDS, 0.2% bromophenol blue, 20% glycerol, 5% 2-mercaptoethanol) and boiled for 5 minutes. Protein samples were run on Bis-Tris (Novex) gels and transferred to nitrocellulose. Blots were blocked in 50% Seablock blocking buffer (Thermo Scientific, diluted in PBS) and incubated with primary antibody overnight in blocking buffer. Antibodies used were to HCC1/RBM39 (Atlas, HPA001591), SIN3B (Santa Cruz, sc-768), Phospho-SMAD1/5 (Ser463/465) (Cell Signaling Technologies, 9516) and beta tubulin (Sigma T5283). Secondary antibodies used were IRDye 680RD Donkey anti-Rabbit IgG and IRDye 800CW Donkey anti-Mouse IgG (LI-COR), incubated for 1 hour in blocking buffer. Blots were imaged on a LI-COR Odyssey CLx. Nuclear and cytoplasmic fractions were extracted with an NE-PER kit (Thermo Scientific), to the manufacturer’s instructions. Separation of cellular fractions was confirmed by blotting for anti-Histone H3 (Abcam, 131711). Phosphorylated and unphosphorylated fractions from C2C12 cells were extracted with a PhosphoProtein Purification Kit (Qiagen) to the manufacturer’s instructions.

### Immunofluorescence

Cells were fixed with ice cold methanol, blocked in 5% BSA and stained overnight with Phospho-SMAD1/5, followed by Goat anti-Rabbit IgG (H + L) Alexa Fluor 488 (Life Technologies) for 1 hour. Cells were counterstained with Hoechst 33342 and imaged on a PerkinElmer Operetta.

### Bioinformatic and statistical analyses

RNA-seq data was mapped using Tophat2 v2.0.12 (https://ccb.jhu.edu/software/tophat/index.shtml) to mouse genome GRCm38/mm10 and ENSEMBL annotated (ENSEMBL release 75). Potential PCR duplicates were removed with MarkDuplicates command part of Picard tools v1.92 (http://broadinstitute.github.io/picard). Gene expression and exon usage were determined by counting uniquely mapped reads with HTSeq-count and dexseq-count.py respectively (http://www-huber.embl.de/users/anders/HTSeq/doc/count.html), with default parameters for both tools. Differentially expressed genes between RBM39 siRNA and non-target control siRNA samples were identified with edgeR using FDR < 0.05. Differential exon usage between the samples was determined with the DEXSeq package using FDR < 0.05. Graphs are expressed as mean +/− standard error (S.E.M.). Densitometry of PCR and western blot bands was carried out with Image Lab software (BioRad). Statistical analysis of differences between groups was by one way ANOVA with post-hoc Tukey’s test or Student’s t-test. Analysis was carried out with GraphPad Prism software (GraphPad).

## Additional Information

**How to cite this article**: Faherty, N. *et al.* Negative autoregulation of BMP dependent transcription by SIN3B splicing reveals a role for RBM39. *Sci. Rep.* **6**, 28210; doi: 10.1038/srep28210 (2016).

## Supplementary Material

Supplementary Information
