# Supplementary material for: Negative autoregulation of BMP dependent transcription by SIN3B splicing reveals a role for RBM39
Source: Sci Rep. 2016 Jun 21;6:28210. doi: 10.1038/srep28210 (PMC4914931; doi:10.1038/srep28210)

Supplementary data

Negative autoregulation of BMP dependent transcription by SIN3B splicing reveals a role for RBM39.

Noel Faherty[1,4,*], Matthew Benson[2,4,*], Eshita Sharma[2,4], Angela Lee[1,4], Alison Howarth[2,3], Helen Lockstone[2, 4], Daniel Ebner[2,3] & Shoumo Bhattacharya[1,4,†].

[1]Division of Cardiovascular Medicine, Radcliffe Department of Medicine, [2]Nuffield Department of Medicine, [3]Target Discovery Institute, [4]Wellcome Trust Centre for Human Genetics, University of Oxford, Oxford, United Kingdom.

[*]These authors contributed equally to this work.

[†]To whom correspondence should be addressed: Shoumo Bhattacharya, University of Oxford, Wellcome Trust Centre for Human Genetics, Roosevelt Drive, Oxford, OX3 7BN, UK.

Email: shoumo.bhattacharya@well.ox.ac.uk.

Supplementary Table S1*: Summary of functions of identified knocked-down splicing factors increasing BMP4-dependent transcription.*

Gene symbol (mouse)	***Name***	***Role in mRNA splicing/processing***	***Refs.***
Dhx15	DEAH (Asp-Glu-Ala-His) box helicase 15	Disassembly of spliceosome after release of mature mRNA	1,2
Pabpn1	Poly(A) binding protein, nuclear 1	mRNA poly(A) tailing	3
Phf5a	PHD finger protein 5A	Interaction between U2 and ATP-dependent helicases	4
Plrg1	Pleiotropic regulator 1	Splice site selection	5,6
Prpf19	Pre-mRNA-processing factor 19	Ubiquitination of PRPF3 and stabilization of U4/5/6 complex, coupling to RNA polymerase II	7,8
Rbm39	RNA binding motif protein 39	U2AF65 associated intron definition	9-11
Sf3a1	Splicing factor 3A subunit 1	Assembly of mature U2 complex	12,13
Sf3a3	Splicing factor 3A subunit 3		
Snrnp200	Small nuclear ribonucleoprotein 200kDa	Core component of U4/5/6 complex, catalysis of RNA unwinding	14,15

1 Wen, X., Tannukit, S. & Paine, M. L. TFIP11 interacts with mDEAH9, an RNA helicase involved in spliceosome disassembly. *Int J Mol Sci* **9**, 2105-2113, doi:10.3390/ijms9112105 (2008).

2 Yoshimoto, R., Kataoka, N., Okawa, K. & Ohno, M. Isolation and characterization of post-splicing lariat-intron complexes. *Nucleic Acids Res* **37**, 891-902, doi:10.1093/nar/gkn1002 (2009).

3 Kuhn, U. *et al.* Poly(A) tail length is controlled by the nuclear poly(A)-binding protein regulating the interaction between poly(A) polymerase and the cleavage and polyadenylation specificity factor. *J. Biol. Chem.* **284**, 22803-22814, doi:10.1074/jbc.M109.018226 (2009).

4 Hubert, C. G. *et al.* Genome-wide RNAi screens in human brain tumor isolates reveal a novel viability requirement for PHF5A. *Genes Dev.* **27**, 1032-1045, doi:10.1101/gad.212548.112 (2013).

5 Ajuh, P., Sleeman, J., Chusainow, J. & Lamond, A. I. A direct interaction between the carboxyl-terminal region of CDC5L and the WD40 domain of PLRG1 is essential for pre-mRNA splicing. *J. Biol. Chem.* **276**, 42370-42381, doi:10.1074/jbc.M105453200 (2001).

6 Lleres, D., Denegri, M., Biggiogera, M., Ajuh, P. & Lamond, A. I. Direct interaction between hnRNP-M and CDC5L/PLRG1 proteins affects alternative splice site choice. *EMBO Rep* **11**, 445-451, doi:10.1038/embor.2010.64 (2010).

7 David, C. J., Boyne, A. R., Millhouse, S. R. & Manley, J. L. The RNA polymerase II C-terminal domain promotes splicing activation through recruitment of a U2AF65-Prp19 complex. *Genes Dev.* **25**, 972-983, doi:10.1101/gad.2038011 (2011).

8 Song, E. J. *et al.* The Prp19 complex and the Usp4Sart3 deubiquitinating enzyme control reversible ubiquitination at the spliceosome. *Genes Dev.* **24**, 1434-1447, doi:10.1101/gad.1925010 (2010).

9 Schneider, M. *et al.* Exon definition complexes contain the tri-snRNP and can be directly converted into B-like precatalytic splicing complexes. *Mol. Cell* **38**, 223-235, doi:10.1016/j.molcel.2010.02.027 (2010).
10 Sharma, S., Kohlstaedt, L. A., Damianov, A., Rio, D. C. & Black, D. L. Polypyrimidine tract binding protein controls the transition from exon definition to an intron defined spliceosome. *Nat Struct Mol Biol* **15**, 183-191, doi:10.1038/nsmb.1375 (2008).
11 Dowhan, D. H. *et al.* Steroid hormone receptor coactivation and alternative RNA splicing by U2AF65-related proteins CAPERalpha and CAPERbeta. *Mol. Cell* **17**, 429-439, doi:10.1016/j.molcel.2004.12.025 (2005).
12 Brosi, R., Hauri, H. P. & Kramer, A. Separation of splicing factor SF3 into two components and purification of SF3a activity. *J. Biol. Chem.* **268**, 17640-17646 (1993).
13 Behrens, S. E., Tyc, K., Kastner, B., Reichelt, J. & Luhrmann, R. Small nuclear ribonucleoprotein (RNP) U2 contains numerous additional proteins and has a bipartite RNP structure under splicing conditions. *Mol. Cell. Biol.* **13**, 307-319 (1993).
14 Cvackova, Z., Mateju, D. & Stanek, D. Retinitis pigmentosa mutations of SNRNP200 enhance cryptic splice-site recognition. *Hum. Mutat.* **35**, 308-317, doi:10.1002/humu.22481 (2014).
15 Laggerbauer, B., Achsel, T. & Luhrmann, R. The human U5-200kD DEXH-box protein unwinds U4/U6 RNA duplices in vitro. *Proc. Natl. Acad. Sci. U. S. A.* **95**, 4188-4192 (1998).

Supplementary figure legends

Supplementary Figure S1. Measurement of endogenous BMP target gene expression with siRNA knockdown of mRNA splicing factors.
(A-C) Expression of the BMP target genes *Id1*, *Id2* and *Smad6* by qPCR. Mouse C2C12 cells were transfected with Silencer Select siRNAs for 24 hours followed by treatment with BMP4 for 24 hours, with the exception of a control treated with diluent only (Vehicle). Y-axis shows relative target gene expression (RQ, mean +/- S.E.M., n=3) normalized to the housekeeping gene *Hprt*. **P*<0.05, ***P*<0.01, versus each graphs respective non-target control (NTC) siRNA. (D) Alkaline phosphatase expression in C2C12 cells. Cells were transfected with siRNA for 24 hours, followed by treatment with BMP4 for 72 hours. Y-axis shows BMP4 induced alkaline phosphatase (mean +/- S.E.M., n=3), normalized to total protein. **P*<0.05 versus NTC siRNA. (E-H) *Pabpn1, Phf5a, Rbm39* and *Dhx15* qPCR. Mouse C2C12 cells were transfected with Silencer Select siRNAs for 24 hours followed by treatment with BMP4 for 24 hours. Y-axis shows relative target gene expression (RQ, mean +/- S.E.M., n=3) normalized to the housekeeping gene *Hprt*. ***P*<0.01, ****P*<0.001.

Supplementary Figure S2. pSmad1/5 phosphorylation in C2C12 cells after siRNA knockdown of RBM39.
Mouse C2C12 cells were transfected with RBM39 siRNA or non-target control (NTC) siRNA for 24 hours followed by treatment with BMP4 for the durations shown. Total protein was extracted and blotted for pSmad1/5, RBM39 and Tubulin expression. Representative blot shown for n=3 experiments.

Supplementary Figure S3. Dynamics of pSmad1/5 localisation in C2C12 cells after siRNA knockdown of RBM39.
Mouse C2C12 cells were transfected with RBM39 siRNA or non-target control (NTC) siRNA for 24 hours followed by treatment with BMP4 for the durations shown. Cells were fixed and stained for pSmad1/5 and counterstained with Hoechst 33342. Representative images shown for n=3 experiments.

Supplementary Figure S4. Expression of VEGF isoforms in C2C12 cells with siRNA knockdown of RBM39.
Mouse C2C12 cells were transfected with either non-targeting control (NTC) or RBM39 siRNA for 48 hours. (A) Expression of VEGF isoforms was determined by PCR using primers recognising multiple transcript isoforms (schematic shown on top of panel). (B) The change in transcript isoform ratio was quantified and normalised to *Gapdh*. **P<0.01. *Fwd*, forward primer, *Rev*, reverse primer.

Supplementary Figure S5. Expression of *Rbm39* in RNA sequencing samples and dataset.
Mouse C2C12 cells were transfected with either non-targeting control (NTC) or RBM39 siRNA (OTP SMARTpool) for 48 hours. (A) Expression of Rbm39 in pooled RNA samples used for sequencing by qPCR. Y-axis shows expression normalised to *Hprt*. ***P<0.001 versus NTC. (B) Log2 expression change in *Rbm39* expression in data from RNA sequencing. FDR – false discovery rate.

Supplementary Figure S6. Pathway analysis of BMP/TGFβ signalling superfamily from RNA sequencing data.
All genes in the BMP/TGFβ signalling superfamily with exons changed in expression with false discovery rate <0.05 are shown. Generated with Ingenuity Pathway Analysis.

Supplementary Figure S7. *Sin3b* splicing and mSIN3 complex expression after knockdown of RBM39.
(A) DEXSeq plot of exon usage for the mouse *Sin3b* gene after knockdown of RBM39 versus non-targeting control (NTC) siRNA. All exons are changed with a false discovery rate (FDR) <0.05. (B) Expression of components of the mammalian SIN3 complex from RNA sequencing data. Graph shows Log2 expression change in expression after siRNA knockdown of RBM39. *FDR<0.05, ***FDR<0.001.

Supplementary Figure S8. RBM39 expression in cells treated with BMP4 and with siRNA knockdown.
C2C12 cells were transfected with RBM39 or non-targeting control (NTC) siRNA for 24 hours followed by treatment with BMP4 for the number of hours shown. Y-axis shows *Rbm39* expression normalised to *Hprt* (RQ +/- S.E.M., n=3). ***P<0.001 versus same timepoint with NTC siRNA.

Supplementary Figure S9. Expression, localisation and phosphorylation of RBM39 in C2C12 cells in response to BMP4.
(A) C2C12 cells were transfected with RBM39 siRNA or non-target control (NTC) siRNA for 24 hours, followed by treatment with BMP4 for 24 hours. Nuclear and cytoplasmic extracts were separated and blotted for RBM39, Histone H3 and Tubulin expression. Representative blot of n=3 experiments. (B) C2C12 cells were treated with BMP4 for 1 hour, phosphorylated and unphosphorylated cell fractions were extracted with a PhosphoProtein Purification Kit and blotted for RBM39 to determine phosphorylation status and pSmad1/5 to demonstrate activation of BMP signalling. Blots were also stained with Ponceau Red as an indication of protein loading. Representative blot of n=3 experiments.

Supplementary Figure S1

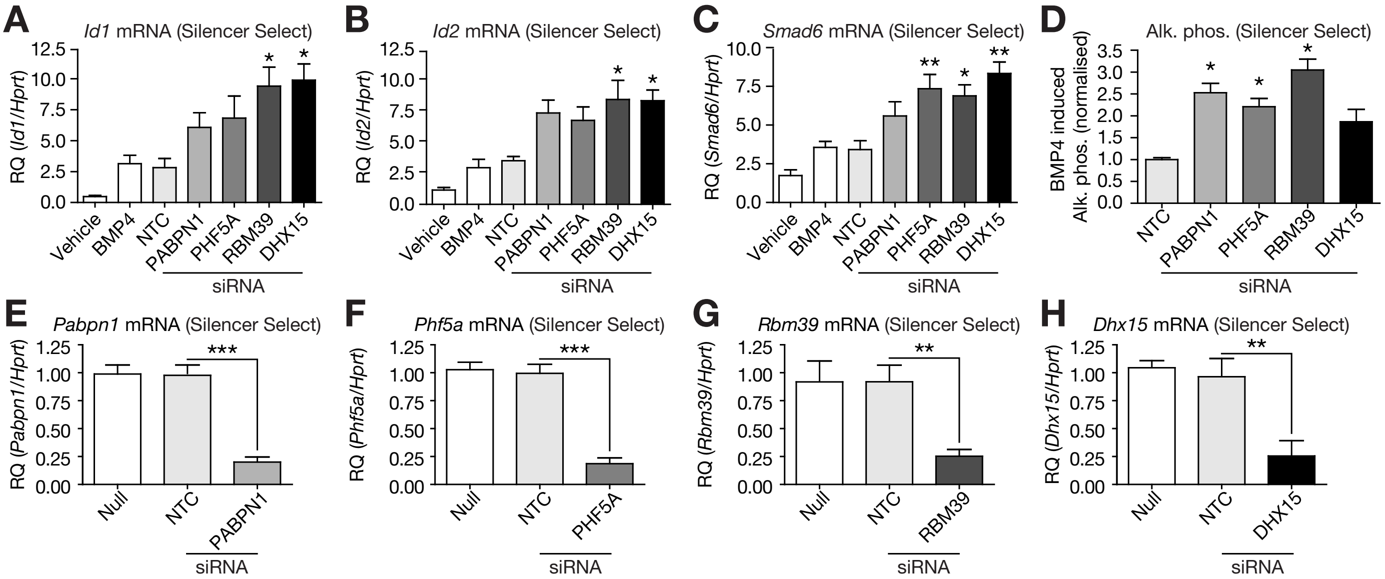

Supplementary Figure S2

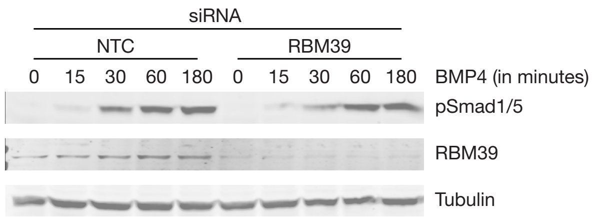

Supplementary Figure S3

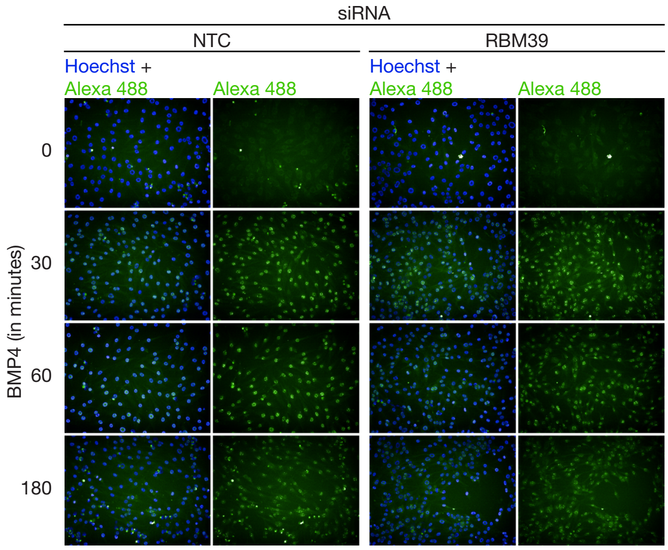

Supplementary Figure S4

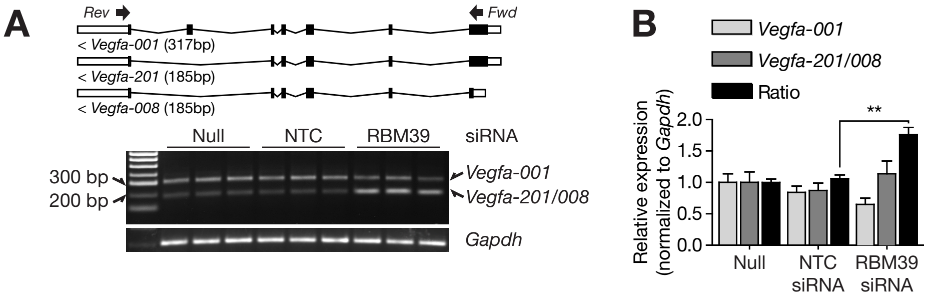

Supplementary Figure S5

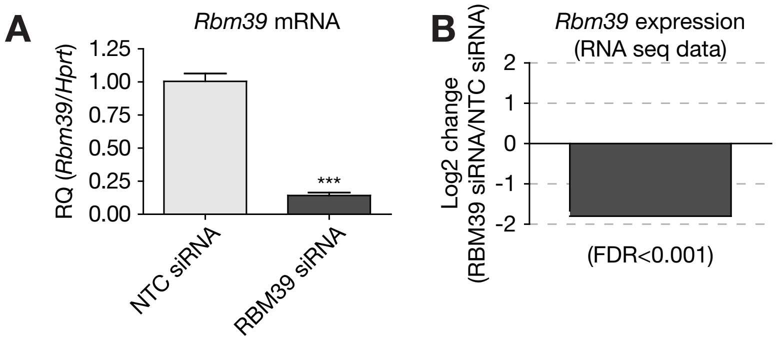

Supplementary Figure S6

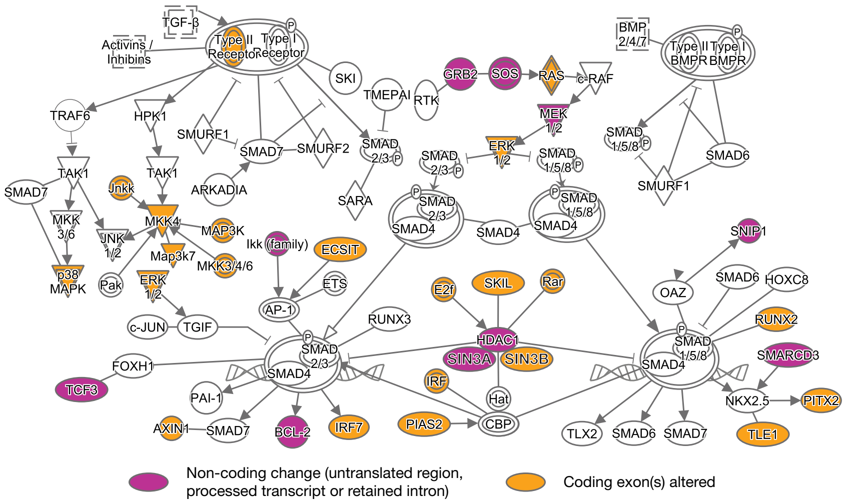

Supplementary Figure S7

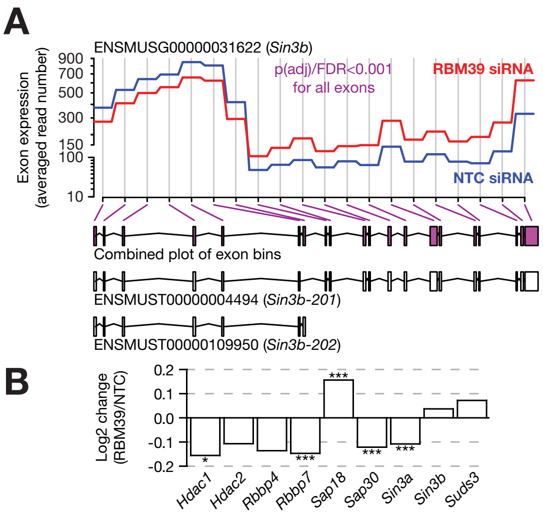

Supplementary Figure S8

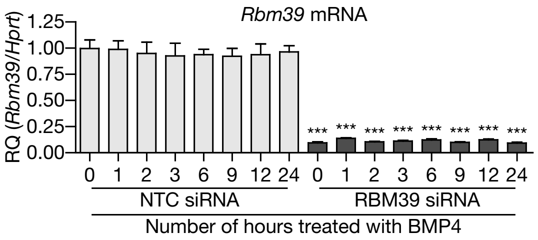

Supplementary Figure S9

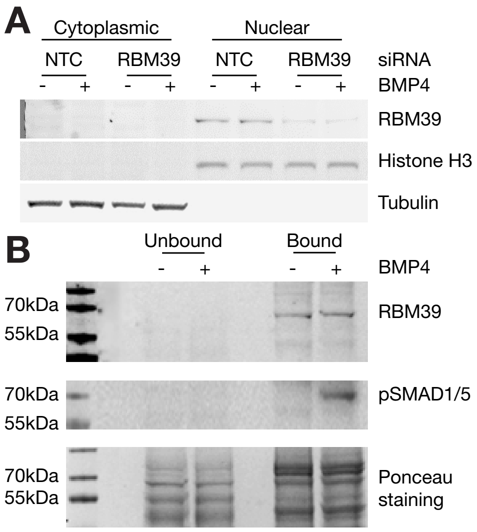

Supplement: Supplementary Information [file srep28210-s1.pdf]
